# A serosurvey study of hand, foot and mouth disease in healthy children aged 6 to 71 months old in West Bandung and Bandung Region, Indonesia

**DOI:** 10.1186/s12879-025-10453-0

**Published:** 2025-01-27

**Authors:** Rodman Tarigan Girsang, Kusnandi Rusmil, Eddy Fadlyana, Budi Setiabudiawan, Riyadi Adrizain, Rizky Perdana Mulyadi, Arief Budiman, Rona Kania Utami, Behesti Zahra Mardiah, Muhammad Gilang Dwi Putra, Frizka Primadewi Fulendry, Dinda Tiaraningrum Nashsyah, Hadyana Sukandar

**Affiliations:** 1 Child Health Department, Faculty of Medicine, Universitas Padjadjaran/Dr. Hasan Sadikin General Hospital, Bandung, Indonesia; 2 Public Health Department, Faculty of Medicine, Universitas Padjadjaran, Bandung, Indonesia; 3 Faculty of Medicine, President University, Bekasi, Indonesia

**Keywords:** Hand, Foot and mouth disease, EV71, Serosurvey

## Abstract

**Background:**

Hand, foot, and mouth disease (HFMD) is an infectious disease that often affects children under 5 years of age. Over the past 20 years, enterovirus 71 (EV71) has become a major concern among children, especially in the Asia-Pacific region. Currently, there are no data showing the seroprevalence of HMFDs in Indonesia. This study aimed to determine the seroprevalence of antibodies to EV71 infection in rural and urban areas.

**Methods:**

This study was an observational analysis and cross-sectional seroprevalence survey of HFMD in children aged 6 to 71 months. The sampling locations were the Padalarang health centre, which is rural, and the Garuda health centre, which is urban. The total sample included 600 children aged 6–71 months from these two locations. Blood sample testing uses enzyme-linked immunosorbent assays (ELISAs) to identify subjects who are positive for IgG EV71 and the risk factors that may influence it.

**Results:**

In total, 596 subjects (99.3%) were positive for the seroprevalence of EV71 IgG in rural and urban areas. Child age, sex, nutritional status, height/age, immunisation status, parental income, and father’s and mother’s educations were not statistically related in rural and urban areas (*p* > 0.05) because the rate of IgG EV71 seropositivity was very high.

**Conclusion:**

This study revealed that the rate of IgG EV71 HFMD seropositivity in Indonesia, especially in the Padalarang health centre and Garuda health centre, was very high. Further research is needed to investigate HFMD cases because of the lack of attention given to this disease and the need to consider whether immunisation is necessary to prevent HFMD.

**Trial registration:**

This study is registered at clinicaltrials.gov, National Clinical Trial (NCT) No. NCT05637229.

## Introduction

Hand, foot, and mouth disease (HFMD) generally presents with mild symptoms but spreads very quickly [1]. HFMD is an infectious disease that most commonly affects children under the age of 5, although it can also occur in adults [2]. The infection involves areas such as the hands, feet, mouth, genitals, and buttocks. The most common cause of the disease is enterovirus 71 (EV71) [3, 4]. This virus is an unencapsulated single-stranded RNA virus and belongs to the genus Enterovirus in the family Picornaviridae. Enteroviruses are classified into types A, B, C, and D, among others. 5 116 serotypes can infect humans, 23 of which are capable of causing HFMD [3, 5, 6]. 

Compared with adults, children are more susceptible to HFMD, largely because of their closer proximity in group settings such as daycare centres and schools [7]. HFMD outbreaks are commonly associated with these environments, as well as with transmission within families[0.3,7] The disease is particularly prevalent among young children under five years of age, who are more likely to contract and spread the infection [6, 7]. Epidemiological data indicate that boys are disproportionately affected by HFMD, with a ratio of 1.6 boys for every girl infected [6]. This suggests a greater susceptibility or greater exposure risk among boys, although the reasons for this disparity are not fully understood [6]. 

Globally, different genotypes of enteroviruses responsible for HFMD circulate with varying prevalence.[6,]7 Genotypes B and C are widely distributed across many regions, whereas genotypes E and F are more common in Africa. Conversely, genotypes D and G are frequently reported in India [6, 7]. These variations in genotype distribution can influence the pattern and severity of HFMD outbreaks in different geographical areas [6, 7]. In the Asia–Pacific region, EV-A71, a major causative agent of HFMD, was particularly notable for its endemic presence in 1990 [7, 8]. Significant outbreaks of EV-A71 have been reported in countries such as Malaysia, Taiwan, and Singapore [8]. These outbreaks have had substantial public health impacts, prompting heightened surveillance and response efforts [8]. 

For example, studies conducted in Shanghai revealed seasonal patterns of HFMD incidence. In 2017, the highest incidence occurred between September and November, which coincided with the autumn season [8]. In contrast, in 2018, the peak incidence was observed from June to August, during the summer months [8]. These findings highlight the seasonal variability in HFMD incidence, which can be influenced by factors such as weather, school terms, and social behaviours.

In Malaysia, the average annual incidence of HFMD was 94.3 cases per 100,000 people, with a significant increase in cases observed between 2017 and 2018 [6]. This increase underscores the need for ongoing public health measures and monitoring to manage and mitigate outbreaks. In Banjarmasin, Indonesia, a specific outbreak in 2016 reported 18 positive cases of serotype EV71. These data reflect the local impact of HFMD and underscore the importance of region-specific surveillance and response strategies to address the disease effectively [9]. HFMD was classified as an acute respiratory infection (ARI) in Yogyakarta, Indonesia, in 2014, although the exact number of patients was not specified [10]. According to research from the Virology Laboratory Centre for Biomedical and Basic Health Technology at the National Institute of Health Research Centre, 26 reported cases (54%) were caused by enteroviruses, including 3 cases of EV71 (6.25%) [11, 12]. 

Overall, understanding the patterns of HFMD, including genotype distributions and seasonal variations, is crucial for developing targeted prevention and control measures [9]. Enhanced surveillance, timely reporting, and public health interventions are essential for managing and reducing the burden of HFMD, particularly in high-risk populations such as young children in communal settings [10]. Currently, there are no reliable data on the annual incidence of HFMD in Indonesia. Although HFMD is frequently encountered in clinical practice, there has been no specific reporting of this disease. Given this context, we aimed to conduct a serosurvey among children aged 6–71 months in Bandung city and the West Bandung region to generate preliminary data on exposure to EV71, the virus causing HFMD. The objective of this study was to determine the seroprevalence of antibodies against EV71 in both rural and urban areas of West Bandung and Bandung city.

## Methods

### Study design and subjects

We performed an observational and cross-sectional study of healthy children aged 6–71 months in the West Bandung Region and Bandung city, Indonesia, who were sampled from rural and urban primary health centres (PHCs) in these areas. The current study was performed at two recruitment sites in Indonesia, Bandung city (an urban area) and the West Bandung Region (a rural area), as follows: PHCs in the West Bandung Region and Garuda in Bandung city. The location was selected on the basis of population density and proximity to the city and looking at socioeconomic conditions, as well as the strong will of the subjects to participate and the ability and resources to conduct research.

After being informed about the nature of the study, written informed consent was obtained from the parents or guardians of the subjects. The investigator checked the inclusion and exclusion criteria to assess subject eligibility before their enrolment in this study.

### Laboratory procedures

After assessing eligibility and collecting the relevant data from the subjects, a trained phlebotomist or nurse carried out the blood drawing. A total of 2 mL of blood was collected in plain blood collection tubes (with a capacity of approximately 2.0–2.5 mL) for the EV71 IgG enzyme-linked immunosorbent assay (ELISA). The samples were labelled with the study code, participant ID, and date and time of collection before being transferred to the Prodia laboratory for analysis.

The dates and times of collection, transfer, and receipt were recorded, and the samples were processed immediately. Blood samples in clotted tubes were centrifuged at 1000 × g for 15 min at room temperature. The serum was then divided into two aliquots and stored in cryovials: one containing approximately 500 µl of serum as the main sample and the other containing the remainder as a backup.

The serum aliquots were stored in a secured freezer (≤ -20 °C) at the Prodia Central Laboratory in Jakarta until use. The specimens were logged and tracked, with access restricted to authorised personnel only. The temperature during storage was monitored and documented on the appropriate forms throughout the trial.

This study employed the ELISA IgG EV71 test (Beier Beijing, China) to detect EV71 antibody responses. Compared with a control tool, this test has a sensitivity of 99.5% for detecting antibodies in sick individuals, with 99.6% sensitivity and 100% specificity for healthy individuals, as validated by a control tool from Abbott. EV71 IgG antibodies are typically negative from 0 to 96 h after the onset of illness. After 96 h, the positive rate of IgG antibodies gradually increased, whereas that of IgM antibodies decreased, indicating that IgM does not persist for a long period of time and eventually converts to IgG. The cut-off value for the test is calculated as NC + 0.1 (where NC = 0.05), resulting in a cut-off of 0.15. Therefore, an EV71 IgG result is considered positive if it is greater than 0.15 and negative if it is less than 0.15.

### Outcomes

The primary objective of this study was to assess the seroprevalence of antibodies against EV71 by measuring the proportion of individuals who tested positive for EV71-specific IgG. To achieve this goal, this study aimed to determine how widespread EV71 infection is within the population by calculating the percentage of seropositive individuals. This was done with a 95% confidence interval to ensure the reliability of the estimates. The results were presented both overall and segmented by different age groups, taking into account the normal distribution of the data.

Additionally, the study involved detailed analysis and tabulation of patient characteristics. This included calculating percentages, standard deviations, and 95% confidence intervals for various demographic and health-related factors. These factors were examined by age group, sex, comorbidities, and socioeconomic status to provide a comprehensive overview of the population under study. By evaluating these aspects, this study aimed to provide a detailed understanding of the prevalence and distribution of EV71. The findings are intended to contribute to public health strategies and inform preventive measures by highlighting seroprevalence patterns across different demographic groups and regions.

### Statistical analysis

The calculated sample size for estimating a population proportion was determined on the basis of the results of the studies of Ang, LW., Phoon, MC., Wu, Y. et al. using the magnitude of the HFMD seroprevalence (14.3%) [13]. To achieve 3% precision, the study required 523 subjects. Considering a 10% dropout rate, the sample size of this study was increased to 600 subjects. Among these 600 subjects, at least 300 were recruited from each of the two site centres. Quota sampling was used to recruit the participants. The quota required in each age stratum was calculated on the basis of the age distribution of the population. Seroprevalence was calculated at 95% confidence intervals (CIs) for binomial proportions via the Wilson method, and their unpaired difference was calculated via the method described by Newcombe. Seroprevalence data were analysed in the per-protocol population via SPSS software (version 18 for Windows).

## Results

**Fig. 1:**
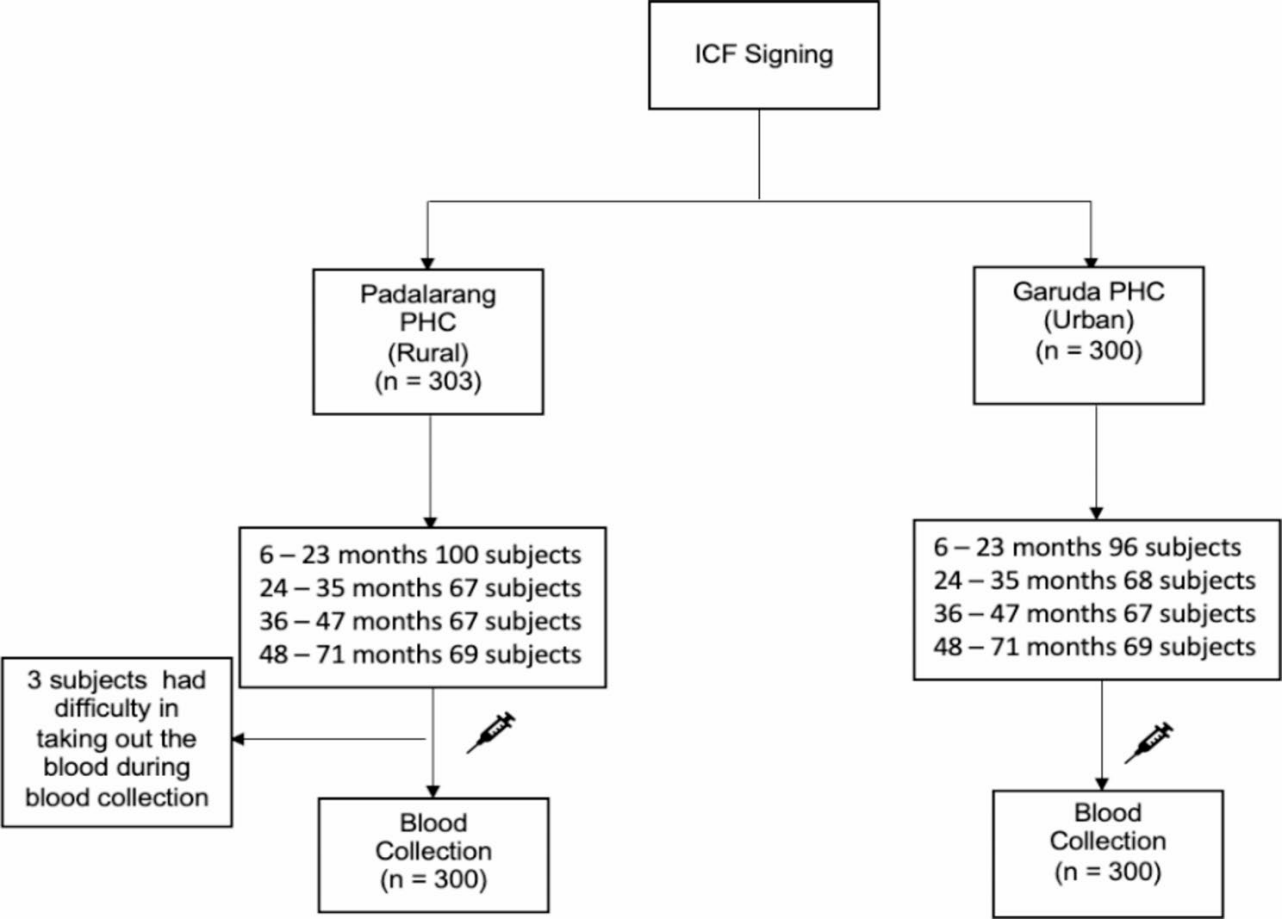
Participant flowchart

Research on the factors influencing the incidence of HFMD in children aged 6–71 months was conducted at the Padalarang Primary Health Centre (representing rural areas) and Garuda Primary Health Centre (representing urban areas). The study took place from November 2022 to January 2023, with 603 subjects recruited through convenience sampling. This included 300 participants from the Garuda Primary Health Centre and 303 from the Padalarang Primary Health Centre. The subjects were divided into four age groups: 6–23 months, 24–35 months, 36–47 months, and 48–71 months (Fig. 1).

The demographic characteristics of the children in the different study areas (see Table 1) reveal that, by age, the largest proportion of children are in the 6–23 month age group, with 32.7% in rural areas and 32.0% in urban areas. In terms of gender, males predominated, accounting for 53.0% of the participants in rural areas and 55.0% in urban areas. Statistically, there was no significant difference between the two areas.

In terms of nutritional status, the majority of children in both rural and urban areas were classified as normal, with 76.3% in rural areas and 83.3% in urban areas. In urban areas, 30.0% of children were in the stunted height/age category. The immunisation rates were notably higher in urban areas (84.0%) than in rural areas (28.0%). Most parents’ incomes in both rural and urban areas fell below the minimum wage, with 61.0% in rural areas and 50.0% in urban areas. The fathers’ highest level of education was predominantly high school, with 54.7% in rural areas and 58.3% in urban areas; similarly, the mothers’ highest level of education was high school (47.3% and 50.7%, respectively). There was a statistically significant relationship between living in rural versus urban areas with respect to nutritional status, height/age, immunisation status, parental income, and the father’s last education, with a p value of less than 0.005.

**Table 1:** Demographic characteristics of the children in the study area

Variable	Rural	Urban	p*
	*n* (%)	*n* (%)	
**Age**			0,992
6–23 Months	98 (32.7)	96 (32,0)	
24–35 Months	65 (21,7)	68 (22,7)	
6–47 Months	68 (22,7)	67 (22,3)	
48–71 Months	69 (23,0)	69 (23,0)	
**Gender**			0,566
Male	159 (53,0)	166 (55,3)	
Female	141 (47,0)	134 (44,7)	
**Nutritional Status**			< 0,001*
Normal	229 (76,3)	250 (83,3)	
Wasted	50 (16,7)	8 (2,7)	
Severely wasted	7 (2,3)	3 (1,0)	
Risk of overweight	7 (2,3)	32 (10,7)	
Overweight	3 (1,0)	5 (1,7)	
Obese	4 (1,3)	2 (0,7)	
**Height for Age**			< 0,001*
Normal	278 (92,7)	196 (65,3)	
Stunted	19 (6,3)	90 (30,0)	
Severely stunted	3(1,0)	14 (4,7)	
**Immunisation Status**			< 0,001*
Completed	84 (28,0)	252 (84,0)	
Under immunisation	216 (72,0)	48 (16,0)	
**Parent’s Income**			0,008*
Above regional minimum wage	112 (37,3)	148 (49,3)	
Below regional minimum wage	183 (61,0)	150 (50,0)	
No income	5 (1,7)	2 (0,7)	
**Father’s Academic Background**			0,001*
Elementary	30 (10,0)	11 (3,7)	
Junior High School	75 (25,0)	61 (20,3)	
Senior High School	164 (54,7)	175 (58,3)	
Bachelor Degree	31 (10,3)	53 (17,7)	
**Mother’s Academic Background**			0,024*
Elementary	38 (12,7)	19 (6,3)	
Junior High School	83 (27,7)	77 (25,7)	
Senior High School	142 (47,3)	152 (50,7)	
Bachelor Degree	37 (12,3)	52 (17,3)	

Note: *) - Chi-square test

- Nutrional status and Height for Age based Regulation of the Minister of Health of the Republic of Indonesia Number 2 of 2020 concerning Child Anthropometry Standards, (2020) [14].

Table 2 provides a detailed overview of the incidence of HFMD across rural and urban areas. The rate of positive IgG EV71 antibodies was 99.3% among the subjects. This high percentage indicates a strong presence of EV71-specific antibodies in the population sampled. The statistical significance of this finding is supported by a p value of 1.00, which suggests that there is no significant difference in IgG EV71 positivity between rural and urban areas.

Additionally, the relative risk (RR) for the incidence of HFMD between the two areas is reported as 1.0, with a 95% confidence interval of 0.987–1.013. This finding indicates that the likelihood of testing positive for IgG EV71 is virtually the same in both rural and urban settings. The confidence interval suggests that the true relative risk is likely to fall within this narrow range, further confirming that there is no substantial difference in the incidence of HFMD between the rural and urban areas studied.

**Table 2:** Positive rate of EV71 IgG in rural and urban areas

Variable	IgG EV71			
	Positive	Negative	p*	RR (CI 95%)
Rural	298 (99,3%)	2 (0,7%)	1,00	1,0 (0,987-1,013)
Urban	298 (99,3%)	2 (0,7%)		

Note: *) Based on Fisher’s exact test

Table 3 presents an analysis of various independent variables related to EV71 infection across rural and urban areas. The table indicates that none of the variables studied—such as age, sex, nutritional status, immunisation status, or socioeconomic factors—had statistically significant associations with the incidence of HFMD in either rural or urban settings. This is reflected by p values exceeding 0.05 for all variables, which means that there is no evidence of a significant relationship between these factors and the risk of HFMD.

In other words, the analysis suggests that the independent variables considered in the study do not significantly influence the likelihood of HFMD occurring in the sampled populations. Therefore, on the basis of the data presented, these variables cannot be identified as risk factors for HFMD in the rural and urban areas studied. This finding implies that other factors not accounted for in this study might have contributed to the incidence of HFMD.

**Table 3:** IgG EV71 analysis based on rural and urban influence variables

**Parent’s Income**			0,531	1,01 (0,99–1,03)			1	1,00 (0,98–1,02)
Above regional minimum wage	112 (100,0%)	0 (0,0%)			147 (99,3%)	1 (0,7%)		
Below regional minimum wage	186 (98,9%)	2 (1,1%)			151 (99,3%)	1 (0,7%)		
**Father’s Academic Background**			1	0,99 (0,98–1,02)			1	1,09 (0,99–1,02)
≤ Junior High School	104 (99,0%)	1 (1,0%)			72 (100,0%)	0 (0,0%)		
≥ Senior High School	194 (99,5%)	1 (0,5%)			226 (99,1%)	2 (0,9%)		
**Mother’s Academic Background**			0,162	0,98 (0,96–1,06)			1	1,01 (0,99–1,02)
≤ Junior High School	119 (98,3%)	2 (1,7%)			96 (100,0%)	0 (0,0%)		
≥ Senior High School	179 (100,0%)	0 (0,0%)			202 (99,0%)	2 (1,0%)		

Note: *) Based on Fisher’s exact test

## Discussion

Hand, foot and mouth disease (HFMD) is a common disease in children [1]. However, there have been no reports of this disease affecting children in Indonesia, particularly in West Java. Previous reports on HFMD were limited to Banjarmasin in 2016, which were based on case findings, and no serosurveys have been conducted in Indonesia [9]. According to demographic data from the West Bandung and Bandung areas, there is no established relationship with the incidence of HFMD. Nevertheless, our research on HFMD serosurveys revealed a notably high number of cases.

Our study demonstrated that there was no significant relationship between age and the incidence of HFMD. This lack of association may be attributed to the very high overall incidence rate observed, which could suggest that the age range of the subjects studied was not a significant risk factor for HFMD infection in this context [5, 9]. This finding is consistent with studies by Esposito S et al., who reported that more than 90% of HFMD cases occur in children under the age of 5 years [5]. 

Furthermore, our research revealed no association between sex and the incidence of HFMD, which aligns with the findings of Liu Z et al., who reported no significant relationship between sex and HFMD incidence [15]. This contrasts with research conducted by Pan et al., which suggested that boys are at greater risk of developing HFMD than girls because of their increased participation in outdoor activities [16]. 

According to Indonesia Basic Health Research, stunting—a condition characterised by impaired growth and development in children due to poor nutrition—is a growing concern in Indonesia. Data from the study indicate a continuous rise in stunting rates from 2010 to 2013, positioning Indonesia as having the second-highest prevalence rate globally, only behind Pakistan. This trend highlights a significant public health issue that requires urgent attention [17]. 

Previous research conducted in Yogyakarta supports these findings, showing that stunting rates remain high in urban areas, which aligns with our study’s observations. The higher prevalence in urban settings may be attributed to various factors, including socioeconomic conditions and access to resources, which can affect nutritional status and overall child development [18]. 

In addition to environmental and socioeconomic factors, genetic predispositions may also play a role in stunting. Genetic factors can influence a child’s growth patterns and nutritional requirements, potentially contributing to the persistence of stunting despite improvements in other areas [19]. Therefore, addressing stunting in Indonesia necessitates a comprehensive approach that considers both environmental and genetic factors to effectively combat this issue [18, 19]. 

Income and parental education level were not related to the incidence of HFMD in our study. In the West Bandung and Bandung areas, both fathers and mothers generally had intermediate levels of education. This level of education could impact parents’ understanding of health and hygiene practices [20]. Moreover, many families have incomes below the minimum wage, which might influence the nutritional status of their children [21]. Poor nutritional status could exacerbate the risk of infections, including HFMD [20–22]. 

These findings contrast with studies conducted in China, where lower socioeconomic status, lower educational attainment of parents or guardians, and reduced household income were found to be associated with more severe cases of HFMD [22]. For example, research by Wang et al. demonstrated that low socioeconomic status significantly affects the severity of HFMD, suggesting that socioeconomic factors play a crucial role in the disease’s impact [22]. Similarly, studies by Kua and Pang, Han et al., and Wang et al. have explored the relationship between socioeconomic factors and HFMD, revealing differing conclusions regarding the influence of these variables on the incidence and severity of the disease [22–25]. 

Kua and Pang’s study in Singapore highlighted epidemiological risk factors for HFMD, noting a potential link between socioeconomic conditions and disease prevalence [23]. Han et al. examined the disease burden of severe HFMD in Jiangsu Province and reported associations between socioeconomic factors and the severity of the disease [24]. Wang et al. investigated the relationship between family affluence and the clinical severity of HFMD among hospitalised paediatric patients in Henan, China, and reported a significant impact of socioeconomic status on the severity of HFMD [25]. 

Overall, while our study did not find a significant relationship between income, parental education, and the incidence of HFMD, existing research from other regions suggests that socioeconomic factors can influence both the prevalence and severity of the disease. These discrepancies highlight the need for further research to better understand how various factors contribute to HFMD and to inform targeted public health interventions [26]. Our study also revealed no relationship between basic immunisation and the incidence of HFMD. This lack of association may be attributed to the high overall incidence of HFMD and the fact that current immunisation programs target diseases specific to the vaccines they offer. In Indonesia, HFMD is often regarded as a mild illness that typically resolves on its own; thus, immunisation is not yet considered necessary [26]. However, the importance of immunisation cannot be overstated, as it plays a crucial role in controlling and reducing diseases that can be prevented through vaccination.

The HFMD vaccine, which is available and has been shown to be necessary, is particularly important because of the potential complications caused by EV71 [27]. Research conducted by Aswathy Raj et al. underscores the importance of vaccines in managing HFMD [28]. For example, a study in Fujian Province, China, reported a significant decrease in the incidence of severe HFMD cases, from 39.02% in 2014 to 11.02%. This reduction highlights the critical role of the HFMD vaccine in controlling the disease and preventing severe outcomes [29]. 

Our research revealed that a total of 596 individuals in the West Bandung and Bandung Regions were infected with EV71. Specifically, there were two individuals in West Bandung (both in the 48–71-month age group) and one individual in each of the 6–23-month and 48–71-month age groups in the Bandung Region who were not infected. The results indicate a high incidence rate of EV71 in both rural and urban areas, even among individuals who did not exhibit symptoms of HFMD. This finding aligns with a 2011 study conducted in Guangzhou, which revealed the presence of EV71 in healthy individuals [30]. Another study revealed that, out of 445 people without any signs or history of HFMD, 53% tested positive for the EV71 virus via an IgG antibody test in the 0–5 year age group [31]. Additionally, a 2007 study in Japan reported that EV71 was isolated in 93.8% of subjects, further highlighting the prevalence of this virus even among asymptomatic individuals [32]. 

Our research revealed no significant difference in the incidence of HFMD between the West Bandung area and Bandung city. This lack of disparity could be attributed to the high population mobility and density in these areas. This observation is consistent with findings from Changchun, China, where urban areas—particularly those on the border between urban and rural zones—exhibit a high risk of disease transmission. In these regions, higher per capita income and the resulting population movements can increase the risk of HFMD because of more frequent human-to-human transmission [33]. 

In contrast, studies conducted in Harbin, Huizhou, and Tokyo show different patterns. In these locations, HFMD rates were higher among rural and suburban children. This increased incidence is often linked to poorer public health conditions, lower standards of personal hygiene and health awareness, and suboptimal nutritional status [34–36]. For example, research by Daniel highlighted that in Indonesia, particularly in East Sumba, household hygiene in rural areas remains underdeveloped, despite its critical role in preventing pathogen transmission [37]. 

Other research conducted by Duan et al. indicated that meteorological factors, including the rainy season, air humidity, and hours of sunshine, are correlated with an increase in HFMD cases [38]. The Bandung area, known for its high levels of rainfall and humidity, may thus be influenced by these environmental factors [39]. However, to date, no research has been conducted in Indonesia specifically examining the relationship between weather conditions and the incidence of HFMD. This gap in research presents an opportunity for further studies to investigate how meteorological factors might affect the prevalence of HFMD in Indonesian settings.

This study used the ELISA IgG EV71 test (Beier Beijing, China) to detect the EV71 antibody response. This method has a sensitivity of 99.5% and a specificity of 100%. According to a study by Wang et al. (2018), the IgG EV71 antibody is typically undetectable from 0 to 96 h after the onset of the disease. After this period, the rate of positive IgG antibodies increases gradually, whereas the level of IgM antibodies decreases, suggesting that IgM antibodies do not persist for a long period of time and eventually convert to IgG. In this study, it was not possible to determine the exact timing of EV71 infection in children, as the study focused solely on measuring IgG EV71 levels, and none of the subjects were clinically diagnosed with HFMD. Research by Huang et al. indicated that HFMD detection should be conducted via PCR for more accurate identification [40, 41]. 

The limitations of this study include the exclusion of several factors known to influence the incidence of HFMD in both rural and urban areas. Factors such as weather conditions, household density, and hygiene practices were not investigated. Additionally, the study was restricted to two regions and focused solely on EV71 as the causative agent of HFMD. Given that HFMD cases have been reported in other areas, further research is warranted to address these gaps and explore additional variables that may affect the incidence of HFMD.

## Conclusion

This study revealed that the incidence of HFMD in Indonesia is significantly greater than previously anticipated, with 99.3% of healthy subjects testing positive for the EV71 virus. The high rate of seropositivity among seemingly healthy individuals underscores the need for increased attention and research into HFMD within the region. Given that this disease has previously received limited attention, it is imperative to conduct further investigations to better understand its prevalence, transmission dynamics, and potential health impacts.

Furthermore, the findings of this study provide valuable insights for public health authorities. The high seroprevalence of IgG EV71 suggests widespread exposure to the virus, which may warrant a reassessment of current health promotion and prevention strategies. The results of this study can serve as a critical reference for the government to enhance health education initiatives and implement targeted interventions aimed at controlling HFMD. By increasing awareness of the high rates of seropositivity and the potential risks associated with HFMD, the government can better address this emerging public health concern and develop more effective strategies to protect vulnerable populations, particularly young children who are at greater risk of the disease.

## Data Availability

Anonymous participant data that underlie the results reported in this article will be available upon completion of the clinical trial. Data requests should be sent to the corresponding author at kusnandi@hotmail.com. The requester must provide a scientifically sound proposal and data transfer agreement for the sponsors’ and collaborators’ approval. Upon approval, the data will be transferred through a secure online platform.
